# Green Urine Discoloration due to Propofol Infusion: A Case Report

**DOI:** 10.1155/2011/242514

**Published:** 2011-12-27

**Authors:** Nobuki Shioya, Yoriko Ishibe, Shigehiro Shibata, Hideyuki Makabe, Shigenori Kan, Naoya Matsumoto, Gaku Takahashi, Yasuhiko Yamada, Shigeatsu Endo

**Affiliations:** Department of Critical Care and Emergency, Iwate Prefectural Advanced Critical Care and Emergency Center, Iwate Medical University, 19-1 Uchimaru, Morioka 020-8505, Japan

## Abstract

We present a 19-year-old man who excreted green urine after propofol infusion. The patient was admitted to our hospital for injuries sustained in a traffic accident and underwent surgery. After starting continuous infusion of propofol for postoperative sedation, his urine became dark green. Serum total bilirubin and urine bilirubin were both elevated. We believe that the green discoloration of the urine was caused by propofol infusion and was related to impaired enterohepatic circulation and extrahepatic glucuronidation in the kidneys.

## 1. Introduction

Green discoloration of urine can be caused by medicines, dyes, infections, ingested substances, and several other factors [1, 2]. Propofol metabolites can discolor urine; however, the pathogenesis of this discoloration is not fully understood. These metabolites are not biologically active substances, and, therefore, green urine associated with propofol is benign [3]. To our knowledge, only 11 cases of propofol-induced green urine have been reported to date [3–13]. We present an unusual case with green urine due to intravenous propofol administration and review the relevant literature.

## 2. Case Presentation

A 19-year-old man was brought to our critical care center for the management of open fractures of the right humerus and radioulna sustained in a major traffic accident. An arteriogram of his right upper extremity showed a right axillary artery injury, and an emergency angiostomy was performed. Anesthesia was induced with administration of propofol, vecuronium, and fentanyl. During the operation, his urine was yellow.

**Figure 1:**
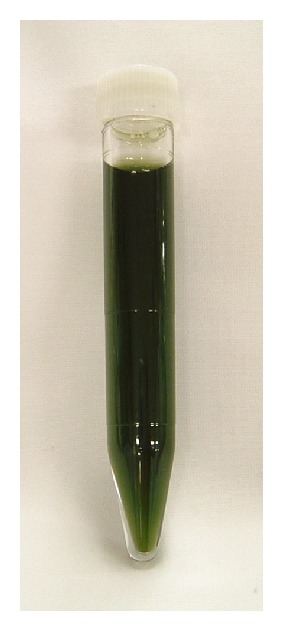
The urine became green after propofol infusion.

 Midazolam, vecuronium, and buprenorphine were used as continuous sedatives for postoperative respiratory management. On day 6 of admission, these sedatives were replaced with 2.0–3.0 mg/kg/h of propofol. At that time, the total bilirubin level was 1.0 mg/dL and the serum albumin level was 1.7 g/dL. For hypoalbuminemia and worsening of anemia (hemoglobin 6.9 g/dL), a 25% albumin preparation and concentrated red cells were administered. After 39 hours of continuous propofol infusion, the patient's urine became dark green (Figure 1). Blood examination showed a total bilirubin level of 2.9 mg/dL, direct bilirubin level of 1.2 mg/dL, serum albumin level of 3.0 g/dL, and hemoglobin level of 11.5 g/dL. An abdominal plane computed tomography (CT) scan showed that his gallbladder was still contrast filled due to constipation. The patient's serum electrolyte and creatinine levels and his white blood cell count were within normal limits. Urinalysis revealed a pH of 7, bilirubin content of 3+, and urobilinogen level over 8 EU/dL. Arterial blood gas analysis showed a pH of 7.46, pO_2_ of 196 mmHg, pCO_2_ of 40.7 mmHg, and HCO_3_ of 28.4 mEq/L. Urine and blood cultures were negative for microbial growth. On day 9 of admission, he had a bowel movement, and the next day his urine was light greenish-yellow. Urinalysis was repeated and the following results were obtained: pH 7, bilirubin content negative, and urobilinogen level over 8 EU/dL. Arterial blood gas analysis showed a pH of 7.41, pO_2_ of 113 mmHg, pCO_2_ of 45.8 mmHg, and HCO_3_ of 28.7 mEq/L. The serum total bilirubin level was 0.8 mg/dL and that of serum albumin 2.9 g/dL. On day 20 of admission, propofol infusion was discontinued and the patient was extubated. 

## 3. Discussion

Green discoloration of urine is an uncommon clinical finding and can be caused by several factors, including medications, consumer products, dyes, or infections [1–3]. We have shown that the main cause of green urine in the present case was the administration of propofol (2,6 diisopropylphenol). Propofol is metabolized not only in the liver and intestine but also in the kidneys. It was assumed that green urine was associated with (1) enterohepatic circulation failure due to constipation and impaired peristalsis, (2) sufficiency of albumin and erythrocytes as carrier proteins due to administration of an albumin preparation and concentrated red cells, and (3) extrahepatic glucuronidation predominantly in the kidneys.

**Figure 2:**
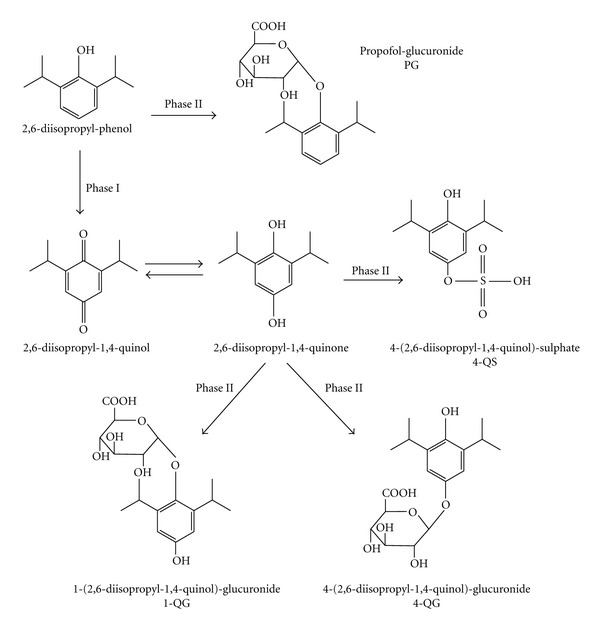
The main metabolic pathway of propofol. In phase I, the metabolism of propofol to 2,6-diisopropyl-1,4-quinol by cytochrome P450 is the rate-limiting step in the formation of 1-QS, 4-QG, and 4-QS. In phase II, the major metabolite was a glucuronic acid conjugate of propofol (propofol-glucuronide) and the minor metabolites were quinol derivatives (1-QG, 4-QG, and 4-QS) [24].

 Propofol is a short-acting intravenous hypnotic agent for anesthesia or sedation. The main metabolic pathway of propofol is oxidation, reduction, and hydrolysis by cytochrome P450 (CYP) and glucuronate conjugation in liver microsomes (Figure 2). CYP2B6 is the predominant CYP isoform involved in the oxidation of propofol by human liver microsomes in phase I [14]. An important group of conjugation reactions are catalyzed by the uridine-5′-diphosphate- (UDP-) glucuronosyltransferases (UGTs) in phase II [15]. UGT1A1 is a relevant isoform in bilirubin glucuronidation. Regarding propofol glucuronidation, a contribution of UGT1A9, which is commonly expressed in the human liver and intestine, has been proposed [16]. However, according to McGurk et al., the glucuronidation of propofol is catalyzed by UGT1A8/9 suggesting higher levels of this isoform in the kidneys than in the liver [17]. Maximum velocity (*V*_max⁡_) for propofol glucuronidation was approximately 3 to 3.5 times higher in the kidneys than in the liver and small intestine.

Propofol was excreted in the urine after glucuroconjugation of the parent drug (to form the propofol-glucuronide) and sulfo- and glucuroconjugation of the hydroxylated metabolite to form 4-(2,6-diisopropyl-1,4-quinol)-sulphate (4-QS), 1-, or 4-(2,6-diisopropyl-1,4)-glucuronide (1-QG and 4-QG), respectively [5, 18]. The urinary rate of propofol metabolites is 68.3% (according to the Japanese literature). The metabolism of propofol to 2,6-diisopropyl-1,4-quinol by CYP is the rate-limiting step in the formation of 1-QG, 4-QG and 4-QS. Propofol-glucuronide is the main metabolite and accounts for approximately 62% of total metabolite profiles. The proportion of these metabolites is 38% (4-QS: 6.7%; 1-QG: 18.1%; 4-QG: 13.2%) [18]. Green urine is caused by these quinol derivatives [2, 4, 5]. These derivatives do not reflect or alter renal function [3].

 Both bilirubin and propofol can bind to human serum albumin [19]. Unconjugated bilirubin is hydrophobic and is transported mainly by albumin in blood [20]. Propofol tightly binds to erythrocytes and serum albumin and is principally bound to albumin (95% bound in pure albumin solutions). Hypoalbuminemia would have an important role in the 25% decrease in binding [21, 22]. It is possible that propofol competes with other medications that bind to the same site in albumin. However, it is unlikely that conjugated bilirubin competes significantly with propofol [24]. In this case, a supplemental albumin preparation and erythrocyte transfusion were given because of hypoalbuminemia (1.7 g/dL) and anemia (hemoglobin 6.9 g/dL) before the urine became green. Thus, it was thought that the delivery rate of propofol was increased. As serum bilirubin rises above 2.0-3.0 mg/dL, conjugated bilirubin is excreted via urine and urinary bilirubin becomes positive. In general, increased levels of urine urobilinogen indicate parenchymal liver damage. However, urine urobilinogen values may be elevated with transit time prolongation, such as with constipation as in this case. It is possible that the degree of propofol glucuronidation in the intestine or liver was lower due to impairment of the enterohepatic circulation by constipation. Furthermore, it was thought that glucuronidation (by UGT1A9) of propofol predominated in the kidneys with the increased capability for propofol delivery secondary to albumin sufficiency. Its determination is affected by urinary pH, and an accurate estimation requires alkalinity. It is well known that propofol metabolites in urine are increased by alkalinization [3, 23]. However, cases with mildly acidic urinary pH have also been reported [4, 8], and in our case neither urine nor blood pH was elevated. A few previous reports have indicated serum total bilirubin to be elevated when green urine was noted [4, 6, 7]. The clinical course in our case is in agreement with the findings of these studies. Although green urine associated with propofol is benign, prompt recognition of this rare effect of propofol may limit unnecessary laboratory tests [3, 4]. 

## 4. Conclusion

The main cause of green urine was considered to be propofol metabolites. Furthermore, we speculate that extrahepatic propofol glucuronidation, predominantly in the kidneys due to impaired enterohepatic circulation secondary to diminished peristalsis, was associated with the appearance of green urine after propofol infusion.
